# Is the Ovarian Cell Pathognomonic?

**Published:** 1882-05

**Authors:** 


### IS THE OVARIAN CELL PATHOGNOMONIC?

Dr. W. A. Edwards (*American Journal Medical Sciences*, April, 1882) considers the question whether a positive diagnosis of ovarian cysts can be made from a microscopic examination of the fluid. He states that he has examined about three hundred fluids. The fluids of ovarian cysts exhibit an abundance of cell-forms of which the epithelial, and a round, granular cell, are most characteristic. The latter, indeed (Drysdale's "ovarian cell"), is said by a certain author to be pathognomonic of ovarian growths. Every-day experience, however, fails to confirm this claim. MM. Malassez and Ch. Robin do not support it. The experience of a number of other authorities, among whom are Peaslee, Spiegelberg, Walter F. Atlee, Braxton Hicks, and Playfair, fails to substantiate the individuality of the ovarian cell. In a case occurring in the practice of Dr. Walter F. Atlee, in which the most typical ovarian cells were present in large numbers, no tumor whatever was found on opening the abdomen.

Thinking that the ovarian cell might be a degenerated pus-cell, Dr. Edwards procured four specimens of laudable pus, two from amputation stumps and two from abscesses, and kept them tightly corked at about the temperature of the body for five weeks. At the expiration of that time, microscopic examination demonstrated the presence of the typical ovarian cell in the pus from the amputation stumps, and what appeared to be the same variety of cell, imperfectly developed, in one of the abscess-specimens.

Dr. Edwards adds the following conclusions :

1. The "ovarian cell" is not diagnostic of the ovarian tumor.

2. We may have a fluid from an ovarian tumor entirely devoid of the ovarian cell.

3. On the other hand, we may have an abdominal fluid which is not ovarian, presenting the cell in great abundance.

4. With the present state of our knowledge, the accurate microscopical diagnosis of ovarian dropsy is impossible ; the most distinguished ovariotomists always making their first incision an exploratory one. The "ovarian cell" is met with so frequently, however, in the fluid from ovarian tumors, that while it is not to be considered as pathognomonic, it still merits some weight in making our diagnosis of ovarian cystomata.

